# The Role of Vaspin in the Development of Metabolic and Glucose Tolerance Disorders and Atherosclerosis

**DOI:** 10.1155/2015/823481

**Published:** 2015-04-06

**Authors:** Rumyana Dimova, Tsvetalina Tankova

**Affiliations:** Department of Diabetology, Clinical Centre of Endocrinology, Medical University, 2 Zdrave Street, 1431 Sofia, Bulgaria

## Abstract

In recent years, most research efforts have been focused on studying insulin-sensitizing adipokines. One of the most recently discovered adipokines is vaspin, a visceral adipose tissue-derived serine protease inhibitor. Vaspin levels have been found significantly increased in mice with obesity and insulin resistance. It has been assumed that vaspin serves as an insulin sensitizer with anti-inflammatory effects and might act as a compensatory mechanism in response to decreased insulin sensitivity. Most studies in humans have shown a positive correlation between vaspin gene expression and serum levels, and metabolic syndrome parameters. Vaspin gene expression is influenced by age and gender, and the administration of insulin sensitizers enhances it in mice, whereas the use of metformin decreases serum vaspin levels in humans, probably due to different regulatory mechanisms. Presumably vaspin plays local and endocrine role in the development of initial and advanced atherosclerosis in obese subjects and might be used as a predictor of coronary and cerebrovascular disease. It is believed that vaspin could be regarded as a new link between obesity and related metabolic disorders, including glucose intolerance. The entire understanding of vaspin intimate mechanism of action might enable the development of novel etiology-based treatment strategies, targeting metabolic and glucose tolerance disorders.

## 1. Introduction

Metabolic syndrome (MetS), defined as a set of cardiovascular disease and type 2 diabetes mellitus (T2DM) risk factors occurring together rather than separately, including abdominal obesity, increased fasting plasma glucose, hypertension, and dyslipidemia [1], underlies a number of socially significant diseases. Its relation to visceral obesity has been proven, since body fat distribution largely determines certain metabolic disorders such as insulin resistance, T2DM, and coronary artery disease (CAD) [2]. Visceral adipose tissue (VAT) not only plays the role of fat depot, but also appears to be an active endocrine organ that in the presence of obesity undergoes hyperplastic changes, involved in certain functions. It is responsible for a wide range of physiological processes, reproduction, apoptosis, inflammation, angiogenesis, blood pressure regulation, atherogenesis, coagulation, fibrinolysis, and immune and vascular homeostasis through direct or indirect impact on the regulation of proliferation. This functional diversity is induced by the ability to synthesize and secrete a multitude of enzymes, hormones, growth factors, cytokines, complement factors, and matrix membrane proteins, termed adipokines. Meanwhile, adipose cells express receptors for most of these substances; thereby adipokines implement their local and systemic effect in response to metabolic changes or other external stimuli. In recent years, most research efforts have been focused primarily on studying adipocytes, secreting insulin-sensitizing adipokines, which play a pivotal role in the peripheral insulin resistance and the development of MetS. A great amount of these adipokines have been identified and their contribution to energy and glucose homeostasis has been reported [3–5].

Although the biological mechanisms, underlying the detrimental impact of visceral fat accumulation, remain unclarified, it seems clear that adipokines might be of clinical importance in the treatment of MetS.

## 2. Vaspin: Mode of Action

One of the most recently discovered adipokines is vaspin, a VAT-derived serine protease inhibitor with insulin-sensitizing effects, belonging to the serpin superfamily, clade A (Serpina12). It is found in the VAT of OLETF (Otsuka Long-Evans Tokushima Fatty) rat, an animal model, characterized with central obesity and T2DM [6]. Both circulating and adipocytes vaspin levels have been found significantly increased in OLETF rats at 30 weeks, the period when they reach the peak of obesity and insulin resistance. Uncontrolled diabetes and weight reduction have diminished vaspin expression, whereas the administration of insulin sensitizers, such as pioglitazone, has normalized its expression and serum concentration [6, 7]. Recombinant vaspin administration in DIO (diet-induced obesity) mice has significantly improved their glucose tolerance and insulin sensitivity. This beneficial effect results in normalizing plasma glucose levels and modifying the expression of genes involved in the pathogenesis of insulin resistance, such as resistin, leptin, TNF*α*, glucose transporter-4, and adiponectin. Based on these data it has been assumed that vaspin serves as an insulin sensitizer with anti-inflammatory effects and might act as a compensatory mechanism with target white adipose tissue (WAT), which is activated in response to the decreased insulin sensitivity [6, 7]. Therefore, it could be speculated that vaspin production antagonizes the effects of unknown proteases which disrupt insulin action [8], having an impact similar to other well-studied systems, such as *α*1-antitrypsin and neutrophil elastase [9].

Nakatsuka et al. have indicated that changes in the vaspin gene are responsible for its compensatory effects on the metabolic abnormalities with regard to obesity. They have demonstrated that vaspin-transgenic mice are protected against diet-induced obesity, glucose tolerance impairment, and fatty liver, whereas vaspin-deficient mice develop glucose intolerance due to upregulation of the endoplasmic reticulum (ER) stress markers. Vaspin has been presented as a circulating serpin, which serves as a ligand for a cell-surface receptor complex, GRP78/MTJ-1 in the liver after ER stress-induced translocation to the plasma membrane. Vaspin exerts its anti-inflammatory action through binding to GPR78, a glucose-regulated protein, and the subsequent signals beneficially affect ER stress-induced metabolic disorders [10]. In another study Nakatsuka et al. have demonstrated that vaspin serves as a ligand for a cell-surface GRP78/voltage-dependent anion channel complex in endothelial cells as well and, thus, exerts antiapoptotic, proliferative, and protective effects on vascular walls in rat models with streptozotocin-induced diabetes mellitus [11]. These reactions display the molecular basis of the direct correlation between this adipokine and ER stress responses of endothelial cells and in the presence of obesity [10, 11]. Furthermore, vaspin protects endothelial cells via an inhibitory effect on NF-kB [12].

As vaspin mechanism of action is not fully understood, identifying the protease targets of vaspin inhibitory effects may lead to the development of new treatment strategies for obesity, diabetes, and insulin resistance [13]. The first protease, determined as a vaspin target, is human kallikrein 7 (hK7), inhibited by classical serpin mechanism with high specificity in vitro. Heiker et al. have isolated vaspin-hK7 complexes in human plasma, established coexpression of both proteins in murine pancreatic *β*-cells, and exhibited the ability of hK7 to cleave human insulin within A- and B-chain. Treatment of isolated pancreatic islets with recombinant vaspin results in increased insulin concentration under conditions of glucose stimulation without affecting insulin secretion and a significant improvement in glucose tolerance in C57BL/6NTac and db/db mice, completely dependent on the serpin activity of vaspin and not related to vaspin-mediated changes in insulin sensitivity. This fact has been confirmed by studies applying a hyperinsulinemic-euglycemic clamp technique (HECT). Probably in db/db mice the improved glucose homeostasis is mediated by elevated plasma insulin concentrations 150 minutes after the glucose load, thus supporting the notion of vaspin inhibiting action on hK7-mediated degradation of insulin in the circulation. hK7 inhibition seems to be the most likely underlying physiological mechanism for the compensatory vaspin effects on obesity-induced insulin resistance [14]. Therefore, the complete elucidation of the molecular mechanisms, regulating vaspin, would gain a new insight into the pathogenesis of MetS, and, as a compensatory molecule in this process, a vaspin recombinant protein or vaspin analogs, antibodies, or small molecule agents might be candidates, underlying new therapeutic agents [7, 15].

The exact reasons for the variability in serum vaspin concentrations remain debatable. A difference in the measurement ranges between ELISA and RIA human vaspin systems have been observed [16–18]. Moreover, Teshigawara et al. and Hida et al. have reported subjects with extremely higher serum vaspin levels in Asians and Caucasians, respectively, employing RIA [17, 18]. Teshigawara et al. have described a minor allele sequence (A) of rs77060950 responsible for these elevated levels [17] and Hida et al. have considered genetic factors, as well [18]. Recently, Breitfeld et al. have conducted a genome-wide association study and identified several single nucleotide polymorphisms (SNPs) in the vaspin locus of 14th chromosome associated with serum vaspin levels and inferred that genetic variations are the most likely reason for serum vaspin variations [19].

Serum vaspin concentrations have shown a specific daily profile according to food intake with peak levels in the early morning fasting period and a significant postprandial decrease 2 hours after breakfast, as this trend has remained the same at the other meals during the day, as well, assuming its role in metabolic regulation. Meanwhile no relationship between serum vaspin and cortisol levels and a strong negative correlation between serum vaspin concentrations and serum insulin and glucose levels during the day have been found, which impugns its insulin sensitizing effects [20].

In a review paper Blüher has summarized that vaspin is predominantly localized in mature adipocytes, isolated from different fat depots, and is expressed in skin, hypothalamus, pancreatic islets, and gastric cells as well, while a stromal or vascular endothelial cells' expression has not been established [13].

## 3. Vaspin and Metabolic Syndrome

There is accumulating evidence that WAT-derived cytokines serve as mediators between obesity-related exogenous factors, such as diet and lifestyle, and the molecular mechanisms leading to MetS and inflammation and/or autoimmune conditions. The role of vaspin in these processes is still debatable [21].

Several studies have shown a positive correlation between vaspin gene expression and the components of MetS. Kloting et al. have investigated vaspin mRNA expression as an indicator for obesity and its association with anthropometric and metabolic parameters in VAT and subcutaneous adipose tissue (SAT) samples. Although vaspin has not been detected in WAT in individuals with normal weight, it has been established in both VAT and SAT in obese subjects with T2DM [22, 23]. A significant correlation between vaspin levels and class of obesity, total body fat percentage, insulin resistance, and glucose intolerance has been demonstrated. In addition, subcutaneous mRNA expression of vaspin significantly correlates with waist/hip ratio, immunoreactive insulin, and fasting glucose infusion rate in steady-state condition during HECT. Contrary to the expectations, vaspin mRNA expression has been found only in 23% of VAT and 15% of SAT samples and there has been no significant correlation between visceral vaspin gene expression and VAT and SAT areas [22]. These findings are supported by another study that has reported vaspin mRNA expression predominantly in nonfat cells [24].

González et al. have studied vaspin gene expression regulation in WAT of mice under different physiological (diet, pregnancy, age, and gender) and pathological (gonadectomy, thyroid dysfunction, and growth hormone deficiency) conditions, associated with energy homeostasis and insulin sensitivity disorders. They have found a decline in vaspin levels after fasting, which partially recovered following leptin administration. Systemic metformin treatment not only improves peripheral glucose uptake and insulin sensitivity [25], but also augments vaspin gene expression. The mRNA peak has been reported on the 45th day after birth in both genders, with higher levels in females as compared to males, and its levels have not shown any change during pregnancy. Hypothyroidism and GH deficiency have suppressed vaspin gene expression. The presented results indicate that vaspin gene expression is influenced by age and gender, that administration of insulin sensitizers enhances it, and that pituitary dysfunction exerts a modifying effect on vaspin levels as well [26]. Contrary to the above findings, the use of metformin in humans reduces serum vaspin level [27]. The results of Tan et al. in subjects with polycystic ovary syndrome (PCOS) are similar. They have found a significantly diminished serum vaspin concentration following a 6-month treatment with metformin [28]. Therefore, the regulatory mechanisms responsible for vaspin gene expression are probably different in humans and mice.

Most studies have assigned an important role of vaspin in the development of obesity and MetS, but it is not clear whether it has causative or protective effect in these conditions. Induction of vaspin mRNA expression might be a compensatory mechanism associated with obesity, severe insulin resistance, and the presence of T2DM, but it remains unclear whether there is a link between human serum vaspin levels and markers of insulin sensitivity and glucose or lipid metabolism.

There are a lot of data in the literature supporting the positive correlation between serum vaspin levels and MetS parameters. In adults [23, 29, 30] and children [31, 32] with obesity and T2DM [16] serum vaspin concentration positively correlates with the class of obesity and insulin resistance. In a cohort of young Korean males it has been found that lower physical activity in combination with a higher percentage of total body fat and elevated immunoreactive insulin levels in the fasting state is associated with increased vaspin levels [33]. Choi et al. have established higher vaspin levels in men with MetS as well [34], whereas Esteghamati et al. have detected elevated vaspin levels in the presence of MetS in both genders and assigned vaspin as a predictor for MetS [35]. Some recent studies have shown quite contradictory results, as lower vaspin levels have been recorded in men with MetS and the trend is worsening with increasing the number of components of MetS [36], and no significant difference in vaspin levels has been observed in subjects with newly diagnosed T2DM and MetS as compared to a group without MetS [37].

There is evidence that VAT independently correlates with serum vaspin concentration in the presence of high HOMA-IR and that insulin resistance has an impact on this association [30]. A condition, characterized by insulin resistance, is PCOS. Tan et al. have examined vaspin levels in overweight females with PCOS and established higher levels of circulating vaspin and vaspin mRNA, located in omental adipose tissue, in the presence of PCOS. Glucose significantly increases vaspin concentration in omental fat depots and thus glucose-mediated vaspin regulation has been proven ex vivo [28]. A positive correlation of vaspin with both insulin resistance and C-reactive protein, as a low-grade inflammatory marker, in the presence of metabolic abnormalities has been observed as well [38, 39]. Contrary to the above, there are several studies that have shown no correlation between vaspin and the presence of insulin resistance. In a study, investigating 108 subjects with normal glucose tolerance (NGT), no association between serum vaspin and glucose tolerance and peripheral insulin sensitivity, measured by HECT and HOMA-IR, respectively, has been identified, and no change in vaspin levels even in the presence of fat-induced insulin resistance has been observed. The results obtained have rejected the assumption of vaspin impact on insulin sensitivity in NGT [40]. Auguet et al. have found no significant difference in vaspin levels between women with NGT, with or without obesity as well [41]. The findings of Bashiri et al. also confirm the conception that there is no relationship between vaspin and insulin sensitivity, and no changes in vaspin levels before and after submaximal 30-minute exercise, in adult overweight men, have been found [42].

Several studies have focused on the changes in serum vaspin levels in weight reduction due to lifestyle changes, diet and exercise. It has been found that vaspin concentrations are lower in subjects with BMI < 25 kg/m^2^ and in those with high long-term physical activity, but weight loss due to a sharp increase in exercise provokes rise in vaspin levels [8, 16]. It could be assumed that vaspin acts as a causative agent in the development of obesity and MetS, and higher vaspin levels following physical activity in untrained individuals are probably related to a different regulation in its secretion at rest and after exercise and might represent a transient adaptation mechanism [16]. This phenomenon has been observed in chronic hemodialysis patients as well [38]. The DIRECT study, including 322 subjects put on a 2-year Mediterranean, low-carbohydrate, or low-fat diet, has revealed permanently lowering of vaspin levels, reflecting the long-term effects of the respective diet and lifestyle changes [43]. In a study, evaluating vaspin levels before and after a 12-week program for weight reduction with orlistat, it has been found that vaspin levels are significantly lower in responders, defined as subjects with ≥2% weight reduction from baseline [23], whereas Kim et al. findings have shown no alteration in vaspin levels after a 10-month program of lifestyle modification in subjects with MetS [44]. Additionally, vaspin has correlated with BMI, body weight, waist, and hip circumferences in the presence of high HOMA-IR [23]. Handisurya et al. have explored vaspin levels in extremely obese subjects after laparoscopic intervention (Roux-en-Y gastric bypass-RYGB) inducing acute weight loss. They have detected a reduction in vaspin, leptin, insulin, C-peptide, BMI, HbA1c, and HOMA-IR levels, and variations in vaspin serum concentration positively correlate with HOMA-IR, immunoreactive insulin, C-peptide, HbA1c, and leptin, and the association between vaspin and HOMA-IR remains significant even after unification in terms of RYGB engendered changes in BMI [29]. The positive relation between vaspin and HOMA-IR after weight reduction has been confirmed in PCOS [28]. Further studies are needed to determine whether vaspin is only a biomarker, reflecting changes in insulin sensitivity, associated with weight loss, or is involved in the regulation of glucose homeostasis in humans [29].

## 4. Vaspin and Atherosclerosis

Adipocytokines play an important role in the pathogenesis of both insulin resistance and diabetes and atherosclerosis [4, 45]. It has been reported that VAT accumulation carries a higher cardiometabolic risk than SAT [5, 46].

It is believed that VAT-derived factors, including adipocytokines such as vaspin, play local and endocrine role in the development of initial and advanced atherosclerosis in obese subjects by affecting the endothelium, vascular smooth muscle cells, and macrophages, thus disrupting vascular homeostasis [47–50]. Kobat et al. have established lower vaspin levels in subjects with CAD as compared to controls, and this tendency has been confirmed in a control group with higher systolic blood pressure in comparison to controls with normal blood pressure. Hence, vaspin might be used as a predictor of CAD [51]. Choi et al. have indicated a significant correlation between plasma vaspin concentrations and the presence and severity of coronary stenosis, calculated by Agatston score, in females [34]. The results of Karbek et al. and Esaki et al. have confirmed the positive association between vaspin and coronary atherosclerosis [39, 45]. Aust et al. have investigated 107 subjects with carotid stenosis and measured fasting vaspin level and its relationship to the severity of atherosclerosis, determined via carotid endarterectomy. The research group has detected lower vaspin levels in subjects with carotid stenosis, having undergone an ischemic vascular event in the last 3 months as compared to those with asymptomatic stenosis, and the lowest levels have been found in subjects with the most recent vascular event, whereas circulating vaspin levels have not correlated with the severity of atherosclerosis [52]. Cura et al. have confirmed the absence of association between vaspin levels and the severity of stenosis but have found elevated vaspin levels in subjects with acute ischemic stroke [53]. Controversial results have been reported in two other studies, demonstrating that vaspin levels are significantly lower in subjects with CAD in comparison with healthy controls and correlate with its severity [51, 54]. Al-Azzam et al. have investigated vaspin levels in subjects with T2DM at baseline and after 8 weeks of simvastatin administration and established higher vaspin concentration following treatment. These data support the notion that the pleiotropic effects of statins, cardioprotective and antiatherosclerotic, might be explained with the rise in vaspin levels beyond their lipid lowering effects [55].

## 5. Vaspin and Glucose Tolerance Impairments

As the impact of vaspin gene variants is generally unknown, in MONICA/KORA F3 study, Kempf et al. aimed to explore the importance of SNPs in the vaspin locus of chromosome 14 in the development of T2DM and obesity. Their results have shown a significant correlation between vaspin SNP rs2236242 and T2DM with genotype AA, carrying increased risk of glucose homeostasis disorders, and this association appears to be independent of BMI. Namely, there is a connection between vaspin and glucose metabolism, and vaspin could be regarded as a new link between obesity and related metabolic disorders, particularly diabetes [56].

Data on serum vaspin levels in T2DM are rather conflicting. Ye et al. have reported higher vaspin levels in subjects with T2DM and a positive correlation between vaspin and postprandial blood glucose levels [57, 58], as Li et al. described a lowering effect of continuous subcutaneous insulin infusion on serum vaspin concentrations concomitantly with the beta-cell function amelioration in T2DM [58]. Other studies have found no difference in vaspin levels between subjects with and without glucose abnormalities [16, 59], or recorded lower vaspin levels in the presence of T2DM [27, 60], respectively. Jian et al. have presumed that lower serum vaspin levels might serve as a risk factor for the development of T2DM [60]. Elevated vaspin concentrations have been established in obese subjects with NGT and prediabetes, as well [61].

Some studies have evaluated vaspin concentrations in T2DM taking into account the disease duration. Atya et al. have reported a decline in vaspin levels with increasing the duration of diabetes [61]. Another study of females with newly diagnosed T2DM and T2DM of different duration have confirmed the data on reducing vaspin levels with increasing diabetes duration. Additionally, Feng et al. have established a significant positive correlation between vaspin and HbA1c in both groups, inverse correlation with HOMA-IR in T2DM with different duration of the disease, and positive correlation with BMI and age in healthy controls, as well [59]. Youn et al. have observed that serum vaspin levels are associated with the presence of obesity and impaired insulin sensitivity in subjects with NGT but not in subjects with T2DM [16]. Li et al. have assessed vaspin in subjects with newly diagnosed T2DM, impaired glucose tolerance, and NGT, following an intravenous 2-week insulin infusion. The circulating vaspin levels have been found significantly lower, and insulin sensitivity and glycemic control have been significantly improved in subjects with newly diagnosed T2DM. Changes in vaspin levels have positively correlated with the increase in insulin resistance, calculated indirectly by HOMA-IR [58]. Based on the above data, it might be asserted that vaspin plays an important role in the pathogenesis of T2DM [62].

The relationship between circulating vaspin levels and the presence of chronic complications of T2DM has also been assessed. Gulcelik et al. have demonstrated lower vaspin levels in females with T2DM and good glycemic control as compared to a group with poor glycemic control, and the presence of microvascular complications has been found to further reduce vaspin levels [27]. Li et al. have examined vaspin levels in individuals with T2DM of up to 3-year duration, with or without macroangiopathy. They have found that the concentration of vaspin is raised in subjects with T2DM without carotid plaques as compared to NGT and diminished in subjects with T2DM with carotid plaques in comparison to those with T2DM without plaques. A significant negative correlation between the presence of carotid plaques and serum vaspin levels has been observed in subjects with T2DM of up to 3-year duration of the disease. These data are a prerequisite to believe that probably vaspin is involved in the process of carotid plaque formation in the early stages of diabetes and that increased vaspin production in human adipose tissue in this period may be a compensatory mechanism associated with obesity, severe insulin resistance, and the development of T2DM and, therefore, may serve as a new biomarker and a protective factor for macrovascular lesions [63]. The compensatory capacity of vaspin secretion gradually lessens with the increase in the duration of diabetes or the onset of cardiovascular diseases and aggravation of atherosclerosis, resulting in a slow decrease in vaspin levels, shown in several studies [6, 51, 52, 54].

## 6. Conclusion 

A meta-analysis, encompassing 6 studies including 1826 obese individuals and 11 studies including 1570 subjects with T2DM, provides evidence of higher vaspin levels in obesity and T2DM and emphasizes the pivotal role of vaspin in the progression of metabolic and glucose abnormalities and, thus, its promising potential as a cardiovascular risk marker [64]. Accumulation of additional data in humans will facilitate the entire understanding of vaspin intimate mechanism of action and, thus, might enable the development of novel etiology-based treatment strategies, targeting metabolic and glucose tolerance disorders [65].

## Abbreviations

MetS: Metabolic syndrome
T2DM: Type 2 diabetes mellitus
CAD: Coronary artery disease
VAT: Visceral adipose tissue
OLETF: Otsuka Long-Evans Tokushima Fatty
DIO: Diet-induced obesity
TNF*α*: Tumor necrosis factor alpha
WAT: White adipose tissue
ER: Endoplasmic reticulum
SNPs: Single nucleotide polymorphisms
hK7: Human kallikrein 7
HECT: Hyperinsulinemic-euglycemic clamp technique
SAT: Subcutaneous adipose tissue
PCOS: Polycystic ovary syndrome
HOMA-IR: Homeostatic model assessment of insulin resistance
BMI: Body mass index
NGT: Normal glucose tolerance.
